# Vascular Involvements Are Common in the Branch Arteries of the Abdominal Aorta Rather Than in the Aorta in Vascular Ehlers-Danlos Syndrome

**DOI:** 10.1016/j.cjco.2022.11.001

**Published:** 2022-11-05

**Authors:** Koichi Akutsu, Atsushi Watanabe, Takeshi Yamada, Tomoko Sahara, Sayuri Hiraoka, Wataru Shimizu

**Affiliations:** a Department of Cardiovascular Medicine, Nippon Medical School, Tokyo, Japan; b Division of Clinical Genetics, Nippon Medical School Hospital, Tokyo, Japan; c Division of Clinical Genetics, Kanazawa University Hospital, Ishikawa, Japan; d Support Centre for Genetic Medicine, Kanazawa University Hospital, Ishikawa, Japan; e Department of Gastrointestinal and Hepato-Biliary-Pancreatic Surgery, Nippon Medical School, Tokyo, Japan

## Abstract

**Background:**

Vascular Ehlers-Danlos syndrome (vEDS) is a rare disorder with poor prognosis, owing to associated vascular complications. However, the most prevalent arterial problems in patients with vEDS are not well known.

**Methods:**

We retrospectively examined 20 consecutive patients diagnosed with vEDS and examined their clinical events, image findings, and therapies.

**Results:**

The age at first complication requiring admission was 29 ± 13 years. The observational period was 67 ± 30 months. Of the 20 patients, 17 took celiprolol at final assessment. At the final follow-up, the total number of complications relating to lesions and requiring admission was 16 for pulmonary lesions (8 patients), 16 for bowel lesions (8 patients), 5 for tendon/ligament lesions (2 patients), 18 for the branch arteries of the abdominal aorta (10 patients), 2 for the aorta (2 patients), and 7 for other arteries (6 patients). Of 54 arterial involvements (aneurysms, dissections, and ruptures), both with and without symptoms, 43 (80%) were in branches of the abdominal aorta (celiac artery and branches, 8; superior mesenteric artery, 4; renal arteries, 3; iliac arteries and branches, 28), 2 (4%) were in the aorta, and 9 were in other arteries. The diameter of the sinus of Valsalva was 29 ± 5 mm, within the normal range. During follow-up, 3 patients died due to suspected ruptures in a branch of the celiac artery, the superior mesenteric artery, and the aorta.

**Conclusion:**

Our findings indicate that lesions involving the branch arteries of the abdominal aorta, rather than aorta, were the most prevalent lesion type in patients with vEDS.

## Résumé

**Contexte:**

Le syndrome d’Ehlers-Danlos vasculaire (SEDv) est une affection rare au pronostic sombre en raison des complications vasculaires qui y sont associées. Toutefois, les problèmes artériels les plus fréquents chez les patients atteints de SEDv sont mal connus.

**Méthodologie:**

Nous avons analysé de manière rétrospective les cas de 20 patients consécutifs ayant reçu un diagnostic de SEDv. Les données relatives aux manifestations cliniques, les résultats des examens d’imagerie et les traitements prescrits ont été examinés.

**Résultats:**

L’âge auquel la première complication nécessitant une hospitalisation est survenue était de 29 ± 13 ans. La période d’observation était de 67 ± 30 mois. Parmi ces 20 patients, 17 recevaient du céliprolol lors de l’évaluation finale. Lors de la dernière visite de suivi, le nombre total de complications associées aux lésions et ayant nécessité une hospitalisation comprenaient : 16 lésions pulmonaires (8 patients), 16 lésions intestinales (8 patients), 5 lésions tendineuses ou ligamentaires (2 patients), 18 complications touchant les ramifications (artères) de l’aorte abdominale (10 patients), 2 complications aortiques (2 patients) et 7 complications d’autres artères (6 patients). Sur les 54 atteintes artérielles (anévrismes, dissections et ruptures), qu’elles aient été symptomatiques ou pas, 43 (80 %) concernaient des ramifications de l’aorte abdominale (artère cœliaque et ses ramifications, 8; artère mésentérique supérieure, 4; artères rénales, 3; artère iliaque et ses ramifications, 28), 2 (4 %) concernaient l’aorte, et 9, d’autres artères. Le diamètre du sinus de Valsalva était de 29 ± 5 mm, soit dans les limites de la normale. Au cours de la période de suivi, 3 patients sont décédés en raison de ruptures suspectées d’une ramification de l’artère cœliaque, de l’artère mésentérique supérieure et de l’aorte.

**Conclusion:**

Les résultats de nos analyses indiquent que les lésions touchant des ramifications de l’aorte abdominale, plutôt que de l’aorte elle-même, étaient les types de lésions les plus fréquents chez les patients atteints de SEDv.

Vascular Ehlers-Danlos syndrome (vEDS) is a type of heritable connective tissue disorder, similar to other such disorders, including Marfan syndrome (MFS) and Loeys-Dietz syndrome (LDS). vEDS is caused mainly by a *COL3A1* mutation that results in increased fragility in the connective tissues of the arteries, bowels, and uterus. In particular, arterial complications have been reported as determinants of prognosis in patients with vEDS.^1^ However, the relative prevalence of arterial lesions and the management practices for patients with vEDS have not been fully clarified. This study aimed to clarify which arterial lesion is most prevalent in vEDS and the care practices for patients with vEDS in daily clinical practice.

## Methods

In this single-centre retrospective study, we analyzed the clinical records of 20 consecutive patients who had been referred to our institute and diagnosed with vEDS.

The vEDS diagnosis was confirmed by identifying *COL3A1* mutations (19 patients) and a decreased type III collagen protein level (1 patient). We looked for *COL3A1* gene variants by screening the entire coding region. Briefly, we extracted genomic DNA from peripheral blood leukocytes and performed high-resolution melting curve analysis to screen for gene variants,^2^ followed by polymerase chain reaction-directed sequencing to ascertain the variant types. In some cases, reverse transcription polymerase chain reaction was performed using total RNA extracted from skin fibroblasts. The *TGFBR1* and *TGFBR2* genes were also investigated as potential mimics of *COL3A1*. As a result, we elucidated the following variant types in 19 patients with *COL3A1* gene mutations: 6 missense mutations that changed a glycine residue to another amino acid, 9 splice site mutations (point mutations at splice junctions that disrupted a splice donor except in 1 case) with additional confirmation for abnormal mRNA synthesis *in vitro* caused by exon skipping, and 4 nonsense mutations caused by base substitutions in stop codons. Of the 19 patients with genetic variants, the levels of type III collagen protein were measured for 4 cases. The genetic variants were evaluated and classified in accordance with the American College of Medical Genetics (ACMG) evaluation system. ^3^ As a result, the pathogenicity of 19 variants was classified as follows: 11 genetic mutations were registered in ClinVar and evaluated by ACMG (pathogenic, 7; likely pathogenic, 4); 7 genetic mutations were registered in databases other than ClinVar and evaluated by ACMG (pathogenic, 4; likely pathogenic, 3); and details of the genetic mutations in the remaining 1 case were unknown, although the amino acid substitution of glycine was recorded and evaluated to be pathogenic. Of the 20 patients in the present study, 18 were probands (individuals who triggered the identification of a family with a hereditary disease) of 18 different families. The remaining 2 patients were related to 2 different probands (ie, they were identified after family screening).

First, we examined the characteristics of the patients. These included the following: age at first complication requiring admission; age at referral to our institute; age at final visit to our institute; gender; family history; follow-up periods; celiprolol intake; number of patients with some complications requiring admission; and death. Second, we examined the number of complications requiring admission due to arterial rupture or dissection with respect to lesion, such as the number of affected patients, the number of patients at first complication requiring admission, the total number of complications at each lesion that required admission, the total number of complications per patient at lesion, and therapeutic strategies for complications. Finally, we examined the pathologic lesions with arterial involvement, including aneurysm, dissection, and rupture. These examinations included asymptomatic patients and incidental findings through imaging at final assessment.

For arterial branches of the abdominal aorta other than the iliac arteries, aneurysms were defined as those > 10 mm. For iliac arteries, aneurysms were defined as those > 15 mm. This is because in patients with vEDS, aneurysm rupture occurs at a smaller diameter than in healthy individuals. The diameter of the sinus of Valsalva was measured and was compared to the normal range as determined by age and body surface area.^4^ Body surface area was calculated using the Mosteller formula.^5^ The diameters of the ascending and descending aorta were measured at the level of pulmonary artery bifurcation, and the diameter of the abdominal aorta was measured at the level of the celiac artery, as reported previously.^6^^,^^7^

The Ethics Committee of our Institute Council approved this study. Data collection was announced on our institute website, and potential participants were given the opportunity to decline further access to their data (opt-out method).

Continuous variables were expressed as the mean ± standard deviation. All statistical analyses were performed using SPSS for Windows, version 27.0 (SPSS, Chicago, IL).

## Results

The characteristics of 20 patients with vEDS are listed in Table 1. The ages at first complication requiring admission and at final visit to our institute were 29 ± 13 years and 42 ± 11 years, respectively. The observational period for outpatients of our institute was 67 ± 30 months. In total, 17 of 20 patients took celiprolol. Of these, 71% took 400 mg of celiprolol daily, at a maximum dose at final assessment.

**Table 1:** Characteristics of the patients

Total number of patients	20
Age, y	
At first event requiring admission	29 ± 13
At referral to our hospital	36 ± 12
At final visit to the hospital	42 ± 11
Male	10 (50)
Family history	11 (55)
Follow-up period, mo	67 ± 30
Reason for diagnosis	19 *COL3A1* mutations 1 decreased type III collagen
Celiprolol intake at final assessment	17 (85)
Daily dose at final assessment, mg/d	324 ± 125
Patients taking 400 mg daily	12 (71)
Patients with complications requiring admission	18 (90)
Deaths	3 (15)

Values are n (%) or mean ± standard deviation.

The number of complications requiring admission for each lesion is shown in Table 2. The most common lesions that required admission were in the branch arteries of the abdominal aorta (18 lesions in 10 patients), accounting for 67% of 27 total arterial complications requiring admission. Among 27 arterial complications, 7 were treated with emergency endovascular therapies (stent-grafting, 1; stent implantation, 2; embolization, 4), and 1 with craniotomy. A total of16 pulmonary lesions (8 patients) and 16 bowel lesions (8 patients) occurred. The number of complications per patient was highest among those with tendon and ligament lesions (2.5). We did not find any uterine complications in our study population. Among 10 female patients, 7 experienced 12 deliveries, including 3 caesarean sections. Only one patient delivered via prophylactic caesarean section, after a vEDS diagnosis.

**Table 2:** Complications related to lesion and requiring hospital admission

Pathologic lesion	Affected patients, n	First complication, n	Total complications, n	Complications /patients	Therapy (incidence)
Arterial branch of the abdominal aorta	10	4	18	1.8	Embolization (4) Stent (1)
Aorta	2	1	2	1.0	Stent-graft (1)
Other arteries	6	3	7	1.2	Stent (1) Craniotomy (1)
Pulmonary	8	7	16	2.0	VATS (2) Pneumonectomy (1)
Colon/intestine	8	3	16	2.0	Surgical repair (16)
Tendon/ligament	2	0	5	2.5	Surgical repair (5)
Uterus	0	-	-	-	-

VATS, video-assisted thoracic surgery.

A total of 54 arterial involvements (aneurysm, dissection, and rupture of arteries—including asymptomatic patients and incidental findings through imaging at final assessment) are listed in Table 3. Arterial involvements were observed in 43 branch arteries of the abdominal aorta (80%; celiac artery, 2; common hepatic artery, 2; splenic artery, 4; superior mesenteric artery (SMA), 4; renal arteries, 3; iliac arteries and their branches, 28) in 12 patients, 2 (4%) in the aorta (2 patients), and 9 (17%) in other arteries (6 patients). The diameter of the sinus of Valsalva was 29 ± 5 mm at final assessment, and no dilatation beyond the normal range occurred. Moreover, the thoracic aorta and abdominal aorta were not dilated. Overall, branch arteries of the abdominal aorta accounted for 80% of the 54 cases with arterial involvement and were the most common arteries for lesion development.

**Table 3:** Pathologic lesion with arterial involvement, with and without symptoms

Pathologic lesions	Affected patients	Details
Age at final assessment, y		41 ± 11
All arterial involvement	14	54
Branch of the abdominal aorta	12 (60)	43 (80) Incidence: celiac (2); common hepatic (2); splenic (4); superior mesenteric (4); renal (3); common iliac (16); external iliac (9); internal iliac (2); jejunal (1)
Aorta	2 (10)	2 (4) Abdominal (1); descending (1)
Height, cm		159 ± 12
Annulo-aortic ectasia, mm	0	Sinus of Valsalva, 28.8 ± 4.7
Thoracic aorticaneurysm, mm	0	Ascending aorta, 29.0 ± 3.6 Descending aorta, 20.5 ± 3.0
Abdominal aortic aneurysm, mm	0	Abdominal aorta, 17.3 ± 2.6
Other arteries	6 (30)	9 (17) Incidence: Intercostal (2); carotid (3); vertebral (1); cerebral (2); coronary (1)

Values are n (%) or mean ± standard deviation, unless otherwise indicated. Arterial involvement (aneurysm, dissection, and rupture) includes asymptomatic patients and incidental findings through imaging at final assessment.

Three patients died during the follow-up period, owing to suspected arterial lesion ruptures. The first patient was 41 years old and suffered cardiopulmonary arrest shortly after complaining about abdominal pain. Autopsy imaging with computed tomography (CT) scans revealed massive hematoma around the collapsed SMA, suggesting rupture of the SMA. The diameter of the SMA was impossible to determine from autopsy imaging, as the SMA collapsed on autopsy imaging (the SMA was 16 mm at 13 months before rupture). The second patient was 35 years old and complained of sudden abdominal pain with shock during hospitalization due to perforation of the descending sigmoid colon and stoma creation. CT scans revealed hematoma near the celiac artery, suggesting rupture of the celiac artery. The diameter of the celiac artery was 14 mm at 5 days before rupture. The third patient was 34 years old and suffered cardiopulmonary arrest shortly after complaining about back pain. Autopsy imaging with CT scans showed massive pleural effusion in the right thoracic cavity linked to hematoma around a collapsed descending thoracic aorta with dissection, suggesting aortic rupture with dissection. The diameter of the descending aorta was 23 mm at 12 days before death. The autopsies of these 3 patients with sudden death were not available.

## Discussion

The main finding of the present study is that in patients with vEDS, arterial involvement is most common in the branch arteries of the abdominal aorta, more prevalent than in the aorta. Moreover, we did not observe dilatation of the aortic root at the level of the sinus of Valsalva, which is one of the characteristic features of MFS.

vEDS, formerly known as Ehlers-Danlos syndrome type IV, is one of the 13 subtypes of Ehlers-Danlos syndrome ^8^ and is caused primarily by *COL3A1* mutations. vEDS is characterized by rupture of the arteries and bowels, and the uterus during pregnancy. The clinical presentation for vEDS is thin, translucent skin of the anterior chest, easy bruising, and small joint hypermobility.^9^ Arterial complications are known determinants of prognosis in patients with vEDS.^1^ From this perspective, vEDS is considered similar to other inherited aortic diseases such as MFS and LDS.

MFS is a representative heritable aortic disease characterized by fragile connective tissues. Common causes of death include aorta-related death, such as that caused by dissection and rupture of aneurysms in the aorta.^10^ The branch arteries of the aorta are also dilated in MFS; however, only 4.8% of these patients were treated during follow-up, and no spontaneous ruptures occurred.^11^ In contrast, in patients with vEDS, complications in the aorta are not as common as they are in those with MFS. One study of patients with vEDS reported that aortic events occurred in 13% of patients, and events in the branch arteries of the abdominal aorta occurred in 12%.^12^ Another study reported that involvement of the aorta and visceral arteries was observed in 24% and 42% of patients with vEDS, respectively.^13^ Further, a study reported that involvement of the aorta and branch arteries of the abdominal aorta accounted for 17.4% and 29.5% of cases with arterial involvement, respectively.^9^ In the present study, pathologic lesions with complications requiring admission, in the aorta and the branch arteries of the abdominal aorta, were observed in 10% and 55% of cases, respectively. In addition, involvement of the aorta and branch arteries of the abdominal aorta accounted for 4% and 80% of 54 arterial involvements, respectively. Therefore, clinicians should focus on the diameter of the arterial branch of the abdominal aorta rather than the aorta in daily medical practice. The low prevalence of aortic complications may be associated with celiprolol intake. In the present study, 85% of patients took celiprolol, which is considered a possible medical therapy for vEDS.^14^, ^15^, ^16^ The reason arterial involvement is more prevalent in the branch arteries of the abdominal aorta is unclear. In terms of the fragility of connective tissues, the arteries of patients with vEDS are thought to be more fragile than those of patients with MFS. If arteries are extremely fragile, branch arteries may be more affected by blood pressure than the aorta, with its richer elastic fiber. As with vEDS, the involvement of the arterial branch of the aorta is also often seen in patients with LDS.^17^

The dilatation of the sinus of Valsalva is known to be common in patients with MFS and LDS^18^; therefore, such dilatation should be carefully observed in patients with MFS and LDS, as dilatation in this region often causes type A aortic dissection and cardiac tamponade, leading to sudden death. However, in the present study, we did not observe any dilatation of the sinus of Valsalva beyond normal ranges as determined by age and body surface area. In patients with vEDS, the sinus of Valsalva possibly is not as dilated as that in patients with MFS, but we were unable to make this comparison in our study.

Therapies for arterial involvement are challenging in patients with vEDS, owing to the extreme fragility of their connective tissue. Clinicians often avoid invasive therapeutic procedures for potentially fatal arterial complications, to the extent possible, as these can lead to iatrogenic complications. Therefore, previous guidelines have not recommended an arterial diameter for prophylactic interventional therapy. We experienced rupture of a celiac artery with a diameter of 14 mm at 5 days before rupture, and a rupture of an SMA with a diameter of 16 mm at 13 months before rupture. Due to our small sample size, we were unable to determine the diameters of branch arteries from the abdominal aorta for interventional therapy. Therefore, we can only comment that invasive therapy should be considered for aneurysms of branch arteries from the abdominal aorta in vEDS patients with smaller arterial diameters than those of healthy individuals. Endovascular therapy is preferred because it is less invasive. In the present study, we found that of 27 arterial complications that required hospital admission, 7 were treated with emergent endovascular therapies, and one with emergent craniotomy. However, none was treated with emergent open thoracic or abdominal surgeries.

The present study has some limitations. First, the most important of these is that the study population was small, which may have caused incidental biases and complications. We should take into consideration that our data may be affected by sampling bias. However, the prevalence of vEDS is assumed to be 1 in 50,000-250,000,^19^ so collection of patient data on a large scale is difficult. Therefore, accumulation of results obtained from even this small population is considered meaningful. Furthermore, our data may be helpful for future meta-analyses. Second, the prevalence of arterial aneurysm depends on its definition. In the present study, aneurysms in the iliac arteries were defined as lesions > 15 mm. This idefinition is stricter than that used in other studies (> 25 mm),^11^ adopting the definition of 1.5 times the normal diameter of the iliac artery in healthy individuals. However, smaller-diameter arterial aneurysms tend to rupture more frequently in patients with vEDS. Therefore, we believe that arterial aneurysms should be defined using a smaller diameter in such patients, in order to prevent arterial dissection and rupture. Third, patients were asked to provide their daily blood pressure records so we could try to maintain their blood pressure levels within their normal range. However, these data were not available. Finally, this study does not provide detailed information on genetic abnormalities in the 19 cases for which genetic testing was performed. However, our aim is to upload our data to the public gene database after obtaining requisite patient consent and ethics committee approval.

## Conclusion

Our data demonstrate that lesions involving the branch arteries of the abdominal aorta were most prevalent in patients with vEDS. In daily clinical practice, clinicians caring for patients with vEDS should pay special attention to the branch arteries of the abdominal aorta rather than to the aorta.
